# Evaluation of the protective effect of quercetin and luteolin against ciprofloxacin- and chloramphenicol-induced oxidative stress in blood cells and their impact on the microbiological activity

**DOI:** 10.3389/fphar.2025.1626058

**Published:** 2025-07-31

**Authors:** Pamela Soledad Bustos, Javier Echeverría, Paulina Laura Páez, María Gabriela Ortega

**Affiliations:** ^1^ Departamento de Ciencias Farmacéuticas, Facultad de Ciencias Químicas, Universidad Nacional de Córdoba, Córdoba, Argentina; ^2^ Instituto Multidisciplinario de Biología Vegetal (IMBIV-CONICET), Córdoba, Argentina; ^3^ Departamento de Ciencias del Ambiente, Facultad de Química y Biología, Universidad de Santiago de Chile, Santiago, Chile; ^4^ Unidad de Investigación y Desarrollo en Tecnología Farmacéutica (UNITEFA-CONICET), Córdoba, Argentina

**Keywords:** ciprofloxacin, chloramphenicol, oxidative stress, flavonoids, protective effect

## Abstract

**Background:**

The induction of oxidative stress (OS) in host cells by antibiotics (ATBs) such as ciprofloxacin (CIP) and chloramphenicol (CMP) is associated with their side effects. Flavonoids such as quercetin (Q) and luteolin (LT) could counteract the harmful effects related to OS induced by ATBs.

**Purpose:**

The purpose of this research was to investigate the *in vitro* effect of CIP and CMP alone and plus Q and LT on ROS production, endogenous antioxidant defenses [superoxide dismutase (SOD) and catalase (CAT)], and protein oxidation (PO) on human leukocytes, evaluating the protective action of Q and LT on the toxicological effects of CIP and CMP.

**Materials and methods:**

Q and LT were isolated from *F. bidentis* leaves and *S. strombulifera* fruits, respectively, and identified by spectroscopic and chromatographic techniques. Cell viability was assessed by the exclusion of the dye trypan blue, and intracellular reactive oxygen species (ROS) were detected by fluorescence using the H2-DCFDA assay. Riboflavin/methionine/NBT and H2O2/dichromate/acetic acid reagents, respectively determined SOD and CAT activities. The advanced oxidation protein products assay was used to assess PO. Q and LT interactions with CIP and CMP were evaluated by checkerboard assay in *S. aureus* and *E. coli*.

**Results and discussion:**

Both ATBs were capable of increasing ROS production in polymorphonuclear cells, and Q and LT were more effective in inhibiting it than vitamin C. Regarding SOD and CAT activity, CIP and CMP altered their activity. Regardless of an increase in enzymatic activity, as in the case of CIP, or a decrease in antioxidant systems, as in the case of CMP, both flavonoids restore enzymatic activity to similar values as those of control cells. Concerning the PO increase observed by CIP and CMP, both Q and LT can prevent it. Finally, the association of flavonoids and ATBs on antimicrobial activity in *S. aureus* and *E. coli* shows antibacterial synergism between LT and both ATBs in the *S. aureus* ATCC strain, thereby enhancing antibacterial activity.

**Conclusion:**

These *in vitro* findings stimulate in vivo research to assess if simultaneous administration of LT/Q with CIP/CMP could be a therapeutic option capable of protecting the host against antibiotic-induced OS in systemic circulation, enhancing antibacterial activity in case of LT. More studies are necessary in order to contribute to this hypothesis.

## 1 Introduction

Some side effects produced by certain antibiotics are related to their ability to increase oxidative stress in various organs, tissues, and cell lines, resulting in metabolic changes that disrupt the body’s normal functioning (Delghandi et al., 2023). Numerous studies suggest that free radical reactions would be involved in the toxic effects of various antibiotics such as gentamicin, clarithromycin, trimethoprim, penicillin, isoniazid, rifampicin, colistin, chloramphenicol, enrofloxacin, and ciprofloxacin on eukaryotic cells (Bustos et al., 2016; Larsen et al., 2017; Biswas et al., 2020; Dumludag et al., 2022; Delghandi et al., 2023; El-Alfy et al., 2024).

Ciprofloxacin (CIP) is the most frequently used fluoroquinolone. It is a broad-spectrum antibiotic commonly used in respiratory, gastrointestinal, osteoarticular, and urinary tract infections. Different studies have been carried out with CIP investigating the involvement of reactive oxygen species (ROS) in the toxicity induced by this antibiotic, relating its phototoxicity, hepatotoxicity, and its effects on the central nervous system with induction of oxidative stress (Gürbay et al., 2007; Ibitoye et al., 2020; Yahia et al., 2020; Badawy et al., 2021). Particularly on human blood cells, it has been shown that CIP is capable of increasing the production of ROS in neutrophils and causing oxidative stress in erythrocytes, generating depletion in the levels of the non-enzymatic antioxidant glutathione (GSH) and conformational and functional changes of the endogenous antioxidant enzyme catalase (CAT) (Becerra et al., 2003; Qin and Liu, 2013). In addition, a study carried out on patients treated with CIP demonstrated an increase in oxidative stress in plasma after 5 days of treatment, manifesting a significant increase of lipid peroxides and a decrease in the antioxidant status, the antioxidant enzyme superoxide dismutase (SOD) and GSH (Talla and Veerareddy, 2011).

Chloramphenicol (CMP) is a broad-spectrum antibiotic used for the treatment of meningitis, bacterial sepsis, typhoid fever, abdominal and bone infections, otitis, pneumonia, and sinusitis; however, it presents toxicity at the level of the hematopoietic system. Thus, although it is a low-cost and highly effective antibiotic, its significant toxicity means that it is not a first-choice antibiotic (WHO, 2021; WHO, 2022). Toxic manifestations of CMP, such as hepatotoxicity, nephrotoxicity, pancytopenia, idiosyncratic aplastic anemia, and skin hypersensitivity, have been attributed to free radical attack, depletion of cellular antioxidants, and induction of lipid peroxidation (Farombi et al., 2002; Popadic et al., 2006; Landge and Kalse, 2021; Delghandi et al., 2023). Because the main side effects of this antibiotic occur in the peripheral circulation, several studies have been conducted on human blood cells. In whole blood, leukocytes and erythrocytes, CMP has been shown to induce oxidative stress, producing an increase in the levels of ROS and reactive nitrogen species (RNS), alteration of endogenous antioxidants, protein oxidation, hemoglobin oxidation, and a decrease in plasma antioxidant potential (Eraso and Albesa, 2007; Páez et al., 2008; Correa-Salde and Albesa, 2009).

Flavonoids are an important group of polyphenols present in plant species with significant antioxidant activity, which is why numerous studies have focused on investigating their ability to counteract the harmful effects associated with oxidative stress. Several antioxidant compounds, such as vitamins and plant extracts, have been evaluated as potential protectors against the toxic effects related to oxidative stress of CMP in rat liver and bone marrow cells and human blood cells. However, to date, there are no studies evaluating the role of isolated flavonoids in the oxidative stress induced by this antibiotic (Farombi et al., 2002; Eraso and Albesa, 2007; Albrecht et al., 2010; El-Alfy et al., 2024). Regarding CIP, the effect of vitamins melatonin, hesperidin, rutin, naringenin, and quercetin on CIP-induced liver, kidney, brain, and heart damage in rats has been evaluated; however, there are no studies evaluating the effect of flavonoids on CIP-induced oxidative stress at the systemic level (Gürbay et al., 2007; Elbe et al., 2016; Shaki et al., 2016; Murad et al., 2019; Al-Naely et al., 2022; Ulutas et al., 2023).

Growing antimicrobial resistance jeopardizes the effectiveness of treating bacterial infections, so the search for natural products capable of counteracting the toxic effects reported by antibiotics is of great importance. Previous studies by our research group demonstrated that the antioxidant flavonoids quercetin (Q) and luteolin (LT) can exert an important protective effect on gentamicin (GEN)-induced oxidative stress in the systemic circulation, with antibacterial synergism between LT and GEN against *Staphylococcus aureus* ATCC 29213 (Bustos et al., 2016; Bustos et al., 2018). Therefore, this work aimed to evaluate the effect of Q obtained from *Flaveria bidentis* (L.) Kuntze [Asteraceae] and LT isolated from *Strombocarpa strombulifera* (Lam.) A. Gray [Fabaceae] (ex *Prosopis strombulifera* var. *strombulifera*) on CIP- and CMP-induced oxidative stress in human leukocytes, by assessing ROS production, endogenous antioxidant defenses, and protein oxidation, to determine whether the protective effect of Q and LT observed in the studies with GEN could be extended to other antibiotics. Furthermore, the effect of the association of each flavonoid with each antibiotic on the antibacterial activity in *Staphylococcus aureus* and *Escherichia coli* was investigated.

## 2 Materials and methods

### 2.1 Reagents and chemicals

Ficoll-Hypaque (Histopaque-1077), trypan blue, 2′,7′-dichlorodihydrofluorescein diacetate (H_2_-DCFDA), nitroblue tetrazolium (NBT), riboflavin, methionine, ethylenediaminetetraacetic acid (EDTA), 1,1,3,3 tetraethoxypropane (MDA), chloramphenicol base (CMP), quercetin (Q) and luteolin (LT) analytical standard were all obtained from Sigma-Aldrich (St. Louis, MO, United States). 2-thiobarbituric acid (TBA) was purchased from Merck (AG, Darmstadt, Germany). Trichloroacetic acid (TCA) was obtained from Fluka Biochemicals (St. Louis, MO, United States), ciprofloxacin hydrochloride (CIP) from Pharafarm (Buenos Aires, Argentina), potassium dichromate from Anedra (Buenos Aires, Argentina), and Chloramine T was obtained from Biopack (Buenos Aires, Argentina). All solvents used were analytical grade.

### 2.2 Plant material

The fruits of *Strombocarpa strombulifera* (Lam.) A. Gray [Fabaceae] (voucher number CORD 1285) and leaves of *Flaveria bidentis* (L.) Kuntze [Asteraceae] (voucher number CORD 2813) were collected in Mendoza and Santa Rosa de Río Primero, Córdoba (Argentina), respectively. The plant material was identified by experts from the Instituto Multidisciplinario de Biología Vegetal (IMBIV-CONICET), and the voucher specimens were deposited at CORD (UNC Botanical Museum). The plant material was dried at room temperature and pulverized.

### 2.3 Extraction, purification, and identification of flavonoids

Quercetin (Q) was isolated from the leaves of *F. bidentis* by acid hydrolysis following previously published methodology (Bustos et al., 2016). Briefly, the sulfate-containing precipitate Q was treated with HCl (2.5 M) and refluxed for 1 h. The precipitate obtained after hydrolysis was subsequently washed with distilled water and partitioned with ethyl ether.

Luteolin (LT) was isolated from *S. strombulifera* fruits, as previously described (Bustos et al., 2018). Briefly, the ethyl ether extract obtained was chromatographed on preparative paper and a Sephadex LH-20 column. LT was obtained from fraction 2.

The identification of quercetin and luteolin was confirmed by spectrophotometric UV–Vis data (Mabry et al., 1970), reversed-phase high-performance liquid chromatography (HPLC) analysis (Bustos et al., 2018), and tandem mass spectrometry (UPLC-MS/MS), and compared with analytical standards (Supplementary Material S1, S2).

For the HPLC, a Varian Pro Star chromatograph (model 210, series 4171), equipped with a reversed-phase column (Phenomenex Hypersil C18, 4.6 × 30 mm) and UV–Vis detector (290 nm) was used. The gradient event of mobile phase solvent A: water (acetic acid 1% v/v) and B: methanol (acetic acid 1% v/v) was as follows: 10%–35% B (10 min), 35%–42% B (15 min), 42%–75% B (10 min), 75% B (5 min), 75%–10% B (5 min), 10% B (5 min), at a flow rate 1.0 mL/min. The injection volume was 20 µL. The retention times of Q and LT were 5.25 min and 5.27 min, respectively. The purity of Q and LT obtained was 96.9% and 97.0%, respectively.

The UPLC MS/MS was performed in Acquity H-Class, Waters, with a quaternary pump equipped with an autosampler and a triple quadrupole mass spectrometer (Xevo TQ-S Micro, Waters). A Waters BEH C-18 (2.1 × 50 mm, 1.7 μm at 30°C) column with an isocratic condition and with the following solvents: solvent A: H_2_O/0.1 v/v formic acid, solvent B: Methanol/0.1 v/v formic acid, 50:50, at a flow of 0.20 mL/min, during 3 min was used for the HPLC conditions. The volume of the injection was 5 μL. All the solutions were filtered through a Millipore filter (0.22 µm).

The MS/MS analyses were performed in negative ion mode, and the other parameters used for compound analysis were: capillary voltage of 3.5 kV, and desolvation gas (nitrogen) at a flow rate of 650 L/h. Desolvation and source block temperature were 350°C and 150°C, respectively.

For the identification of Q and LT by MS/MS, the Multiple Reaction Monitoring (MRM) methodology was used. The monitored mass transitions were 301 > 151 and 301 > 179 for Q, and 285 > 133 and 285 > 131 for LT.

### 2.4 Oxidative stress in blood cells

#### 2.4.1 Leukocyte isolation from human blood

Mononuclear (MN) and polymorphonuclear (PMN) leukocytes were isolated from human blood of healthy volunteer donors (ethics committee approval, CIEIS-HNC 11/16/2018) as previously described (Bustos et al., 2016). MN cells were separated using a Ficoll-Hypaque gradient, followed by hypotonic lysis of red blood cells to obtain PMN leukocytes. Cells were adjusted to 10^6^ cells/mL in Hank’s balanced salt solution (HBSS).

#### 2.4.2 Cell viability

The percentage of leukocyte viability was estimated by exclusion of the vital dye trypan blue. This staining is based on the fact that living cells have intact plasma membranes, so they can exclude trypan blue, whereas dead cells cannot exclude the dye, which is why they appear blue under the microscope (Crowley et al., 2016). Leukocytes (MN and PMN) were incubated for 1, 2, and 4 h with CIP (0.5, 16, and 28 μg/mL in HBSS) or CMP (1, 10, and 50 μg/mL in HBSS). These concentrations were selected according to a curve of ROS production vs. antibiotic concentration, choosing those in which a significant increase in ROS was obtained, also considering that they included the maximum plasma concentration reached after the administration of therapeutic doses of this antibiotic. Subsequently, the same volume of 0.02% w/v trypan blue dye was added, and cell viability was determined by counting in a Neubauer chamber after 10 min of contact between the cell suspension and the dye. The control sample was processed without antibiotic treatment, and its viability percentage was greater than 95%.

#### 2.4.3 Intracellular ROS measurement by fluorescence assay

Intracellular ROS measurement was performed using the H_2_-DCFDA assay as described previously (Bustos et al., 2016). Cells were incubated with CIP (0.5, 16, and 28 μg/mL in HBSS), CMP (1, 10, and 50 μg/mL in HBSS), flavonoids alone or with antibiotic plus flavonoids [10, 50, and 250 µM by diluting the stock solution (1 mg/mL, ethanol) in HBSS, initial concentrations for screening]. The background fluorescence of all compounds tested was corrected by the inclusion of parallel blanks without the fluorescent probe. HBSS was used as a control to evaluate the dose-response relationship of flavonoids at the average concentrations used for each antibiotic (16 μg/mL and 10 μg/mL for CIP and CMP, respectively). The concentrations of Q and LT ranged from 0.05 to 0.63 µM to cover the ROS inhibition range (0%–100%). IC_50_ values (µmolar concentration inhibiting 50% of ROS production) were obtained using OriginPro® 8 (Northampton, MA). Vitamin C was used as the reference inhibitor.

#### 2.4.4 Endogenous antioxidant enzyme activity

The activity of endogenous antioxidant defenses SOD and CAT was estimated as previously described (Bustos et al., 2016). SOD activity was determined using riboflavin/methionine/EDTA/NBT reagents in the presence of light, and CAT activity was measured using H_2_O_2_/dichromate/acetic acid reagents. Leukocytes (10^6^ cells/mL) were incubated for 1 h at 37 °C with CIP, CMP, flavonoids alone, or antibiotics plus flavonoids (Q and LT) at different concentrations. Flavonoid concentrations were selected based on the IC_50_ values calculated for the fluorescence experiment. These concentrations were 0.1, 0.2, and 0.3 µM for Q and 0.1, 0.2, and 0.6 µM for LT. The results were expressed as enzyme units per 10^6^ cells.

#### 2.4.5 Protein oxidation determination by advanced oxidation protein products (AOPP) assay

To evaluate biomarkers of oxidative stress, protein oxidation was performed using an advanced oxidation protein products assay as previously described (Bustos et al., 2021). Cells were treated for 4 h with CIP, CMP, flavonoids alone, or each antibiotic plus each flavonoid at different concentrations (0.1, 0.2, and 0.3 µM to Q and 0.1, 0.2, and 0.6 µM to LT). Chloramine T (0–100 µM) was used as the standard for the calibration curve. Protein content of the samples was determined by Bradford assay (Bradford, 1976). Data were expressed as μmol chloramine T equivalents per mg of protein.

### 2.5 Flavonoids and antibiotics interactions by checkerboard assay in bacteria

Interactions between flavonoids and antibiotics were evaluated as described previously (Bustos et al., 2018) by checkerboard assay on Mueller-Hinton broth following the indications of CLSI (2020). Four strains have been evaluated: two sensitive strains to CIP and CMP (*S. aureus* ATCC 29213 and *E. coli* ATCC 25922), and two clinical strains (*S. aureus* resistant to CIP and *E. coli* resistant to CIP). It should be noted that, given the potency of CMP’s action and the low rate of resistance to this antibiotic, no resistant strains were found. The antibiotic concentrations analyzed in the checkerboards varied according to the minimum inhibitory concentration (MIC) values of each bacterial strain (Bustos et al., 2016; Bustos et al., 2018). The concentrations of each tested agent used in the combinations corresponded to serial dilutions of two-fold their MIC values. The concentrations of CIP tested ranged from 0.004 to 2,048 μg/mL, the CMP concentration from 0.5 to 128 μg/mL, and flavonoid concentrations from 0.25 to 125 μg/mL. When possible, the fractional inhibitory concentration index (FICI) of the combination of each antibiotic plus each flavonoid tested was calculated (Bustos et al., 2018). The FICI value of each agent was calculated by completely inhibiting the growth of the microorganism in the combination well. Based on the FICI value obtained, the type of interaction between flavonoids and antibiotics was determined as synergism, additivity, indifference, or antagonism.

### 2.6 Statistical analysis

Statistical analysis was carried out using the GraphPad Prism program (GraphPad Software, CA, United States). Data was expressed as mean ± S.D. The results were analyzed by one-way analysis of variance (ANOVA) followed by a Tukey test for *post hoc* analysis. A *p*-value <0.05 was considered statistically significant.

## 3 Results

### 3.1 Oxidative stress in human leukocytes

#### 3.1.1 Cell viability and intracellular ROS production

In the cell viability assay of human leukocytes exposed to antibiotics, no alterations in the % of viable cells were observed after exposure of MN cells to CIP and CMP, nor in PMN leukocytes exposed to CIP and to concentrations of 1 and 10 μg/mL of CMP (data not shown). On the other hand, exposure of PMN cells to the maximum concentration of CMP evaluated (50 μg/mL) caused a decrease in cell viability of 18% compared to untreated cells, obtaining a cell viability percentage of 94.9% ± 2.9% for control cells and 76.6% ± 2.5% for cells exposed to 50 μg/mL of CMP.

**FIGURE 1:**
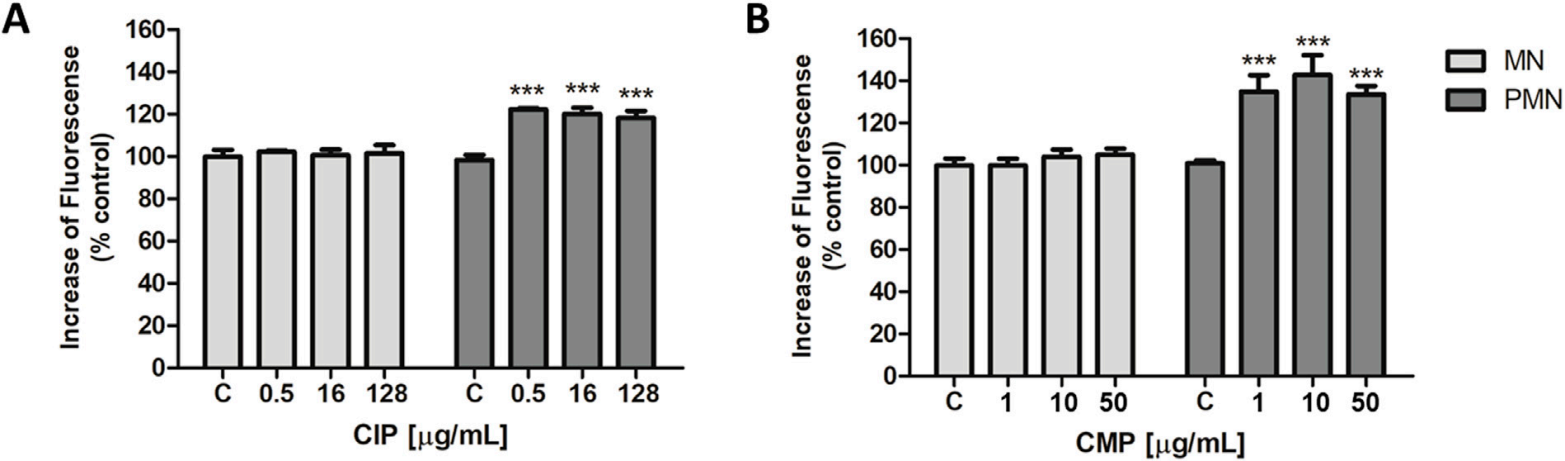
Intracellular ROS production of CIP **(A)** and CMP **(B)** in MN and PMN leukocytes. Data (means ± S.D.) were expressed as percent of values in control cells of three independent experiments. ***p < 0.001 vs. control.

Regarding free radical generation, the first series of experiments examined CIP- and CMP-induced ROS production in human MN and PMN leukocytes. Exposure of MN leukocytes to various concentrations of CIP and CMP did not result in an increase in ROS content compared to untreated control cells (Figure 1).

On the other hand, in PMN cells, ROS levels increased by 21.8 ± 2.0, 20.5 ± 2.7, and 18.6% ± 2.5%, respectively, for the three CIP concentrations evaluated, compared to control cells (Figure 1A). While exposure of PMN leukocytes to different concentrations of CMP produced an increase in ROS production of 36.3% ± 3.7% for the concentration of 1 μg/mL, 44.5% ± 5.4% for 10 μg/mL, and 31.3% ± 0.9% for the maximum concentration evaluated, 50 μg/mL (Figure 1B).

**FIGURE 2:**
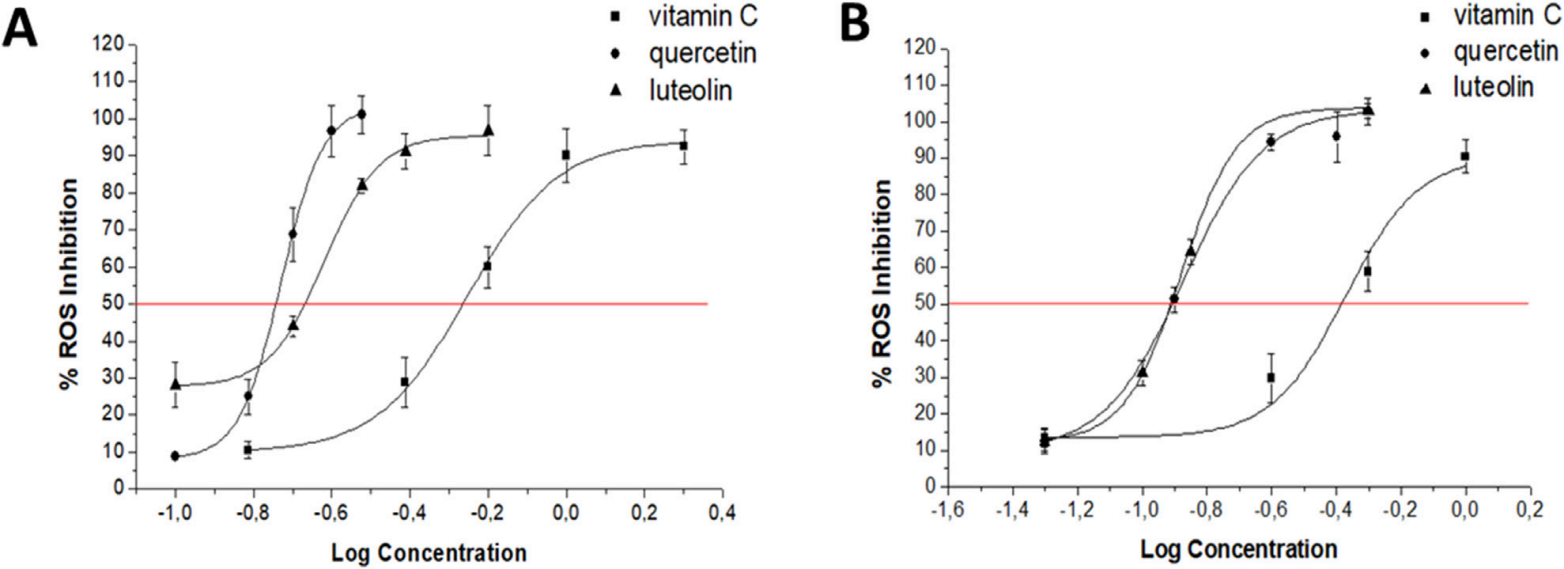
Dose–response curves for Q, LT, and vitamin C on intracellular ROS produced by 16 μg/mL CIP **(A)** and 10 μg/mL CMP **(B)** in PMN human leukocytes.

When evaluating the effect of flavonoids (at selected concentrations) on ROS production induced by CIP (0.5, 16, and 128 μg/mL) and CMP (1, 10, and 50 μg/mL) in PMN leukocytes, both flavonoids showed significant ROS inhibitory activity, reaching 100% inhibition in all cases (Table 1). To compare the activity of flavonoids with each other and with vitamin C (reference inhibitor), five different concentrations of each flavonoid were evaluated in cells exposed to 16 μg/mL of CIP and 10 μg/mL of CMP to estimate their IC_50_ values. Q and LT showed lower IC_50_ values than the reference inhibitor, both for CIP- and CMP-induced ROS. In the case of CIP-induced ROS production, Q showed greater inhibitory activity than LT, since LT showed an IC_50_ value two times lower than that of vitamin C, while the estimated value for Q was three times lower than that of the latter. For CMP-induced ROS production, both flavonoids demonstrated a superior inhibitory effect to vitamin C, obtaining IC_50_ values 3.5 times lower than the reference inhibitor for both flavonoids. At the concentrations evaluated to determine the IC_50_ values, flavonoids did not modify ROS production compared to the basal control (data not shown) (Figure 2; Table 2).

**TABLE 1:** Inhibitory effect of screening concentrations of flavonoids on intracellular ROS induced by CIP and CMP in PMN leukocytes.

Flavonoid concentration (µM)		Inhibition of ROS production (%)					
		Ciprofloxacin (µg/mL)			Chloramphenicol (µg/mL)		
		0.5	16	128	1	10	50
Quercetin	10	102.7 ± 5.0	101.4 ± 9.6	108.2 ± 9.9	102.0 ± 2.4	106.0 ± 2.9	104.0 ± 9.4
	50	107.2 ± 7.5	103.9 ± 2.8	109.5 ± 9.6	106.0 ± 9.2	101.9 ± 4.6	106.1 ± 9.2
	250	102.4 ± 8.8	102.7 ± 9.0	107.1 ± 9.7	100.4 ± 1.1	108.6 ± 6.1	109.9 ± 9.5
Luteolin	10	105.2 ± 9.6	109.4 ± 9.4	102.3 ± 9.4	102.2 ± 9.3	104.9 ± 3.8	107.9 ± 9.4
	50	104.8 ± 4.4	108.3 ± 1.8	109.4 ± 9.8	109.9 ± 3.4	102.7 ± 5.7	104.2 ± 4.9
	250	100.3 ± 9.1	101.8 ± 9.8	109.3 ± 5.8	107.9 ± 9.4	101.2 ± 6.7	103.4 ± 3.4

Data were expressed as percentage inhibition of ROS production by CIP and CMP treatment compared to the control. Each column represents the mean ± S.D. of three independent experiments. No significant difference between means.

**TABLE 2:** IC_50_ values (μM) estimated for flavonoids and the reference inhibitor (vitamin C) on intracellular ROS produced by CIP (16 μg/mL) and CMP (10 μg/mL) in PMN leukocytes.

Compound	IC_50_ (µM)	
	Ciprofloxacin	Chloramphenicol
Quercetin	0.18 ± 0.01^ab^	0.13 ± 0.01^a^
Luteolin	0.23 ± 0.01^a^	0.12 ± 0.01^a^
Vitamin C	0.55 ± 0.01	0.42 ± 0.01

^a^ *p* < 0.001 vs. vitamin C, ^b^ *p* < 0.01 vs. luteolin.

#### 3.1.2 Endogenous antioxidant enzymes activity

**FIGURE 3:**
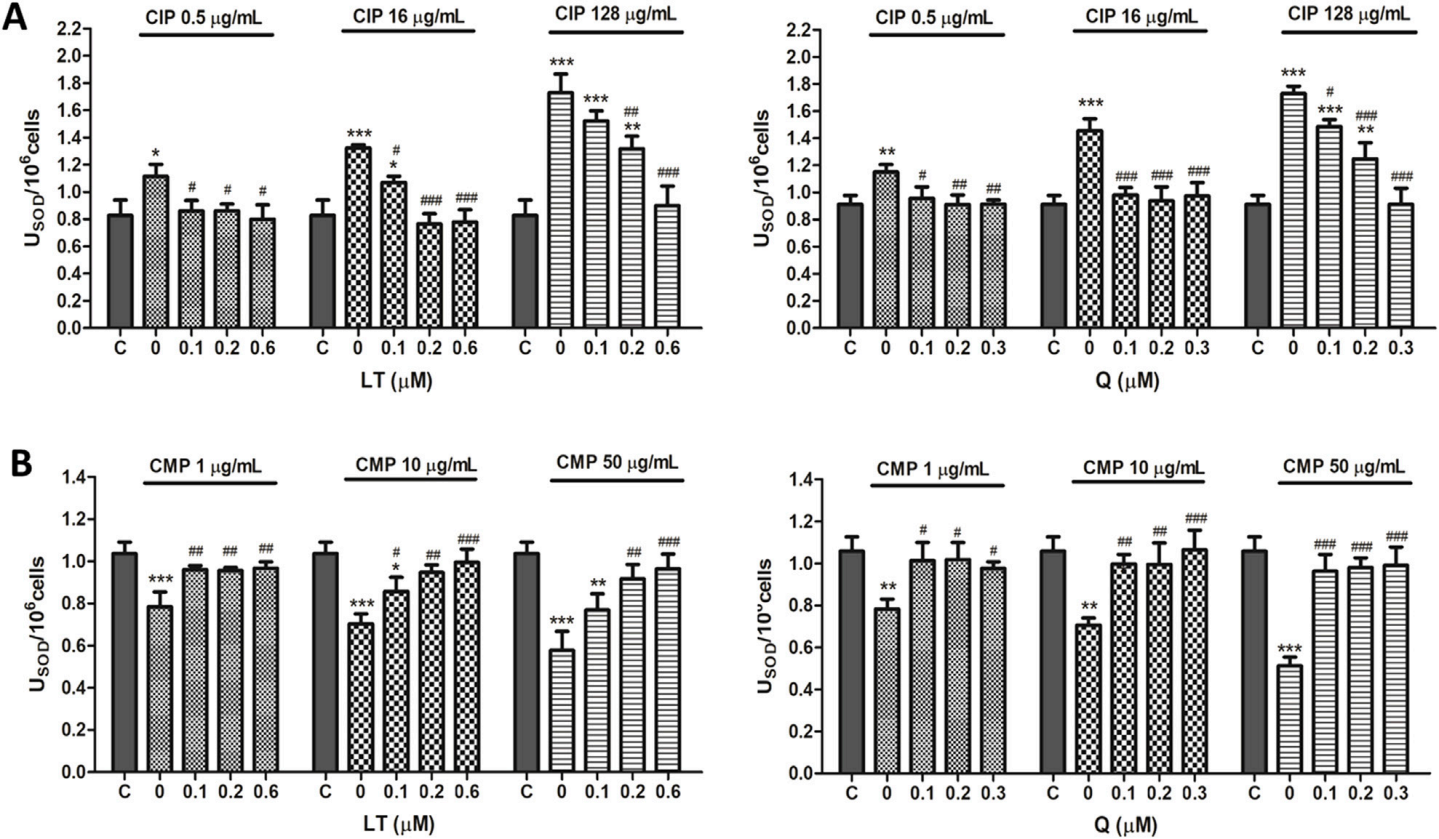
Effect of LT and Q on SOD enzyme activity in PMN human leukocytes exposed to CIP **(A)** and CMP **(B)**. Data (means ± S.D.) are expressed as SOD units per 10^6^ cells of three independent experiments. *p < 0.05, **p < 0.01, ***p < 0.001, statistical differences compared to control leukocytes; #*p* < 0.05, ##*p* < 0.01, ###*p* < 0.001, statistical differences compared to leukocytes treated with CIP or CMP.

**FIGURE 4:**
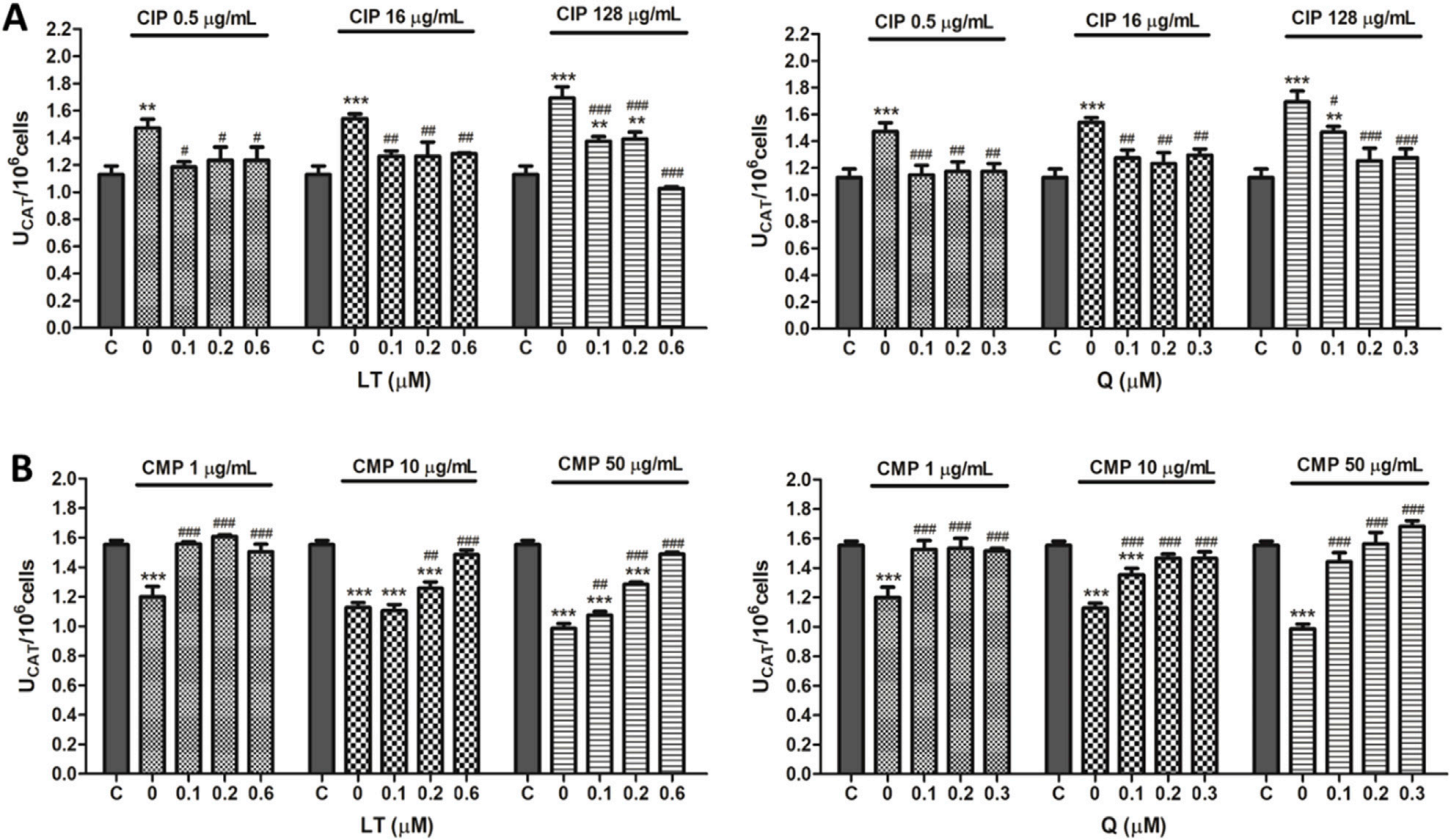
Effect of LT and Q on CAT enzyme activity in PMN human leukocytes exposed to CIP **(A)** and CMP **(B)**. Data (means ± S.D.) are expressed as CAT units per 10^6^ cells of three independent experiments. *p < 0.05, **p < 0.01, ***p < 0.001, statistical differences compared to control leukocytes; #*p* < 0.05, ##*p* < 0.01, ###*p* < 0.001, statistical differences compared to leukocytes treated with CIP or CMP.

In PMN leukocytes, CIP was able to induce an increase in the enzymatic activity of both SOD and CAT (Figures 3A, 4A). SOD activity was increased 30.7 ± 3.7, 58.4% ± 1.5% and 98.9% ± 8.3%, while CAT activity swelled 27.5 ± 2.0, 36.6% ± 1.0% and 50.0% ± 5.2% for concentrations of 0.5, 16, and 128 μg/mL of CIP, respectively. On the other hand, contrary to the effect caused by CIP on enzyme activity, a decrease in SOD and CAT activities was observed after exposure of PMN leukocytes to CMP (Figures 3B, 4B). Exposure of PMN to 1, 10, and 50 μg/mL of CMP caused a 25.2 ± 1.1, 32.8% ± 0.5% and 46.4% ± 2.1% decrease in SOD activity and a 21.4 ± 4.6, 27.4% ± 2.0% and 36.5% ± 2.4% decrease in CAT activity, respectively.

Regarding the effect of the flavonoids LT and Q on the altered activity of the antioxidant enzymes SOD and CAT by CIP and CMP, it was observed that both LT and Q tend to restore the activity of the antioxidant enzymes reaching values similar to control cells (Figures 3, 4). LT was able to restore the enzymatic activity altered by CIP and CMP at all three concentrations tested when cells were exposed to the lowest concentrations of the antibiotics (0.5 μg/mL CIP and 1 μg/mL CMP), whereas at the highest concentrations of CIP and CMP, the effect of LT was concentration-dependent, reaching the values of control cells at 0.6 μM LT. As for Q, it was able to restore the SOD and CAT activity altered by CMP, reaching the values of the control cells at the three flavonoid concentrations evaluated, while for CIP, the co-treatment with the three concentrations of Q maintained the enzymatic activity at values similar to the control cells when the cells were exposed to the lowest and medium CIP concentrations (0.5 and 16 μg/mL). Finally, during exposure of PMN leukocytes to the highest dose of CIP (128 μg/mL), a dose-dependent protective effect of Q was observed, reaching basal values of SOD activity at 0.3 μM of Q and CAT activity at 0.2 μM of the flavonoid. Q and LT alone do not alter the activities of SOD and CAT *per se* at the evaluated concentrations (data not shown).

#### 3.1.3 Oxidative damage to proteins

**FIGURE 5:**
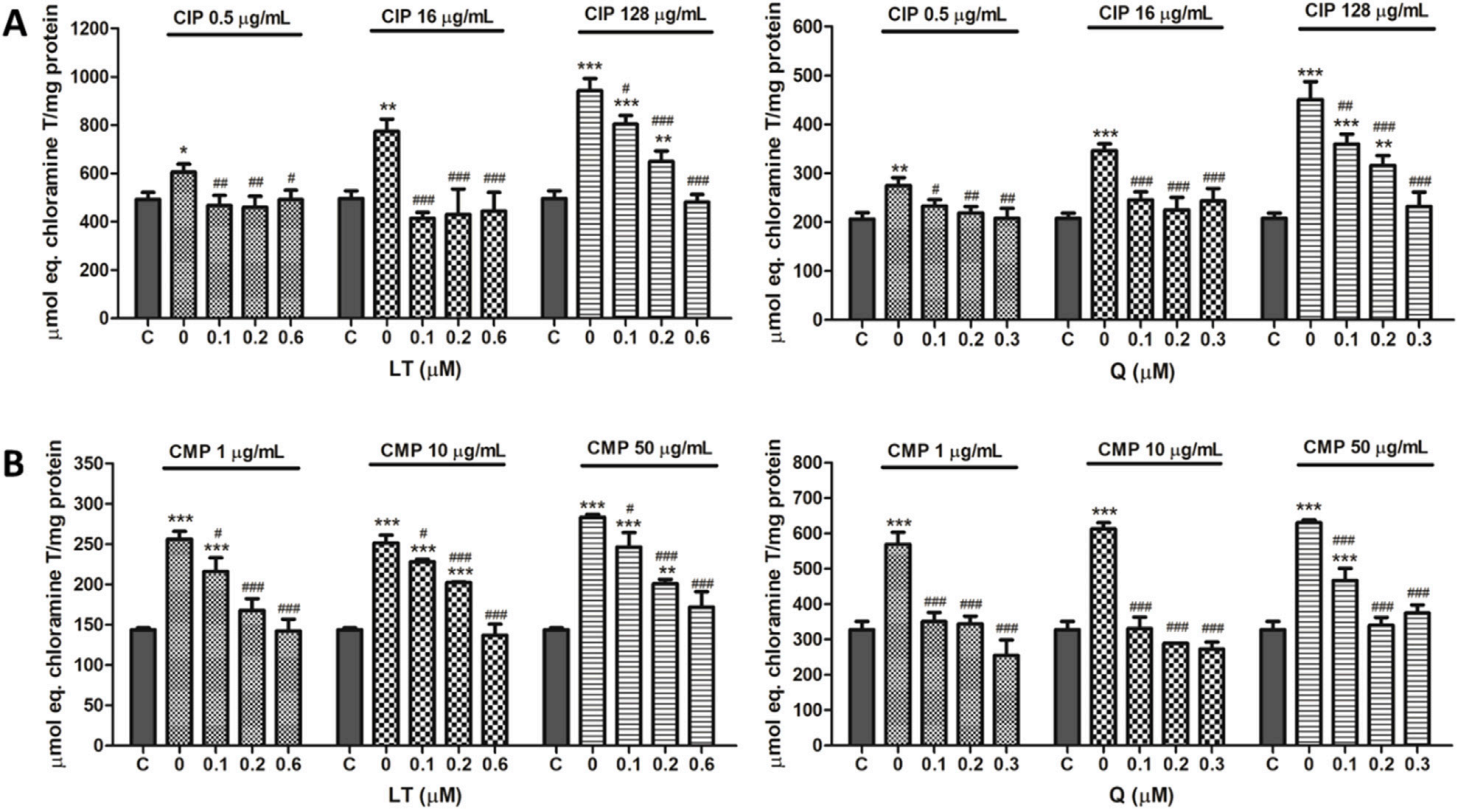
Effect of LT and Q on protein oxidation induced by CIP **(A)** and CMP **(B)** in PMN human leukocytes. Data (means ± S.D.) are expressed as µmol chloramine T per mg protein of three independent experiments. *p < 0.05, **p < 0.01, ***p < 0.001, statistical differences compared to control leukocytes; #*p* < 0.05, ##*p* < 0.01, ###*p* < 0.001, statistical differences compared to leukocytes treated with CIP or CMP.

To evaluate protein oxidative damage as a biomarker of oxidative stress, protein products of advanced oxidation were analyzed. In PMN leukocytes, CIP and CMP treatments increased protein oxidation in a dose-dependent manner (Figure 5). CIP concentrations (0.5, 16, and 128 μg/mL) induced AOPP increases of 26.7 ± 4.4, 61.9 ± 4.6, and 104.2% ± 12.7%, respectively, while CMP treatment (1, 10, and 50 μg/mL) increased AOPP by 73.8 ± 2.1, 80.9 ± 6.1, and 94.7% ± 2.4%, respectively.

Regarding the effect of treatment with LT and Q, both flavonoids prevented protein oxidation induced by CIP and CMP, maintaining values similar to those of control cells. For CIP at 0.5 and 16 μg/mL, both flavonoids prevented protein oxidation at the three concentrations tested, while at the highest concentration of the antibiotic, the protective activity of LT and Q was concentration-dependent. In the case of CMP at the three concentrations of the antibiotic, Q was able to maintain protein oxidation at values similar to those of control cells in all cases, while the protective effect of LT was dependent on the concentration of the flavonoid, reaching the values of control cells at 0.6 μM (Figure 5). Q and LT alone maintain protein oxidation levels similar to control cells (data not shown).

### 3.2 Flavonoids and antibiotics interactions by checkerboard assay in bacteria

**FIGURE 6:**
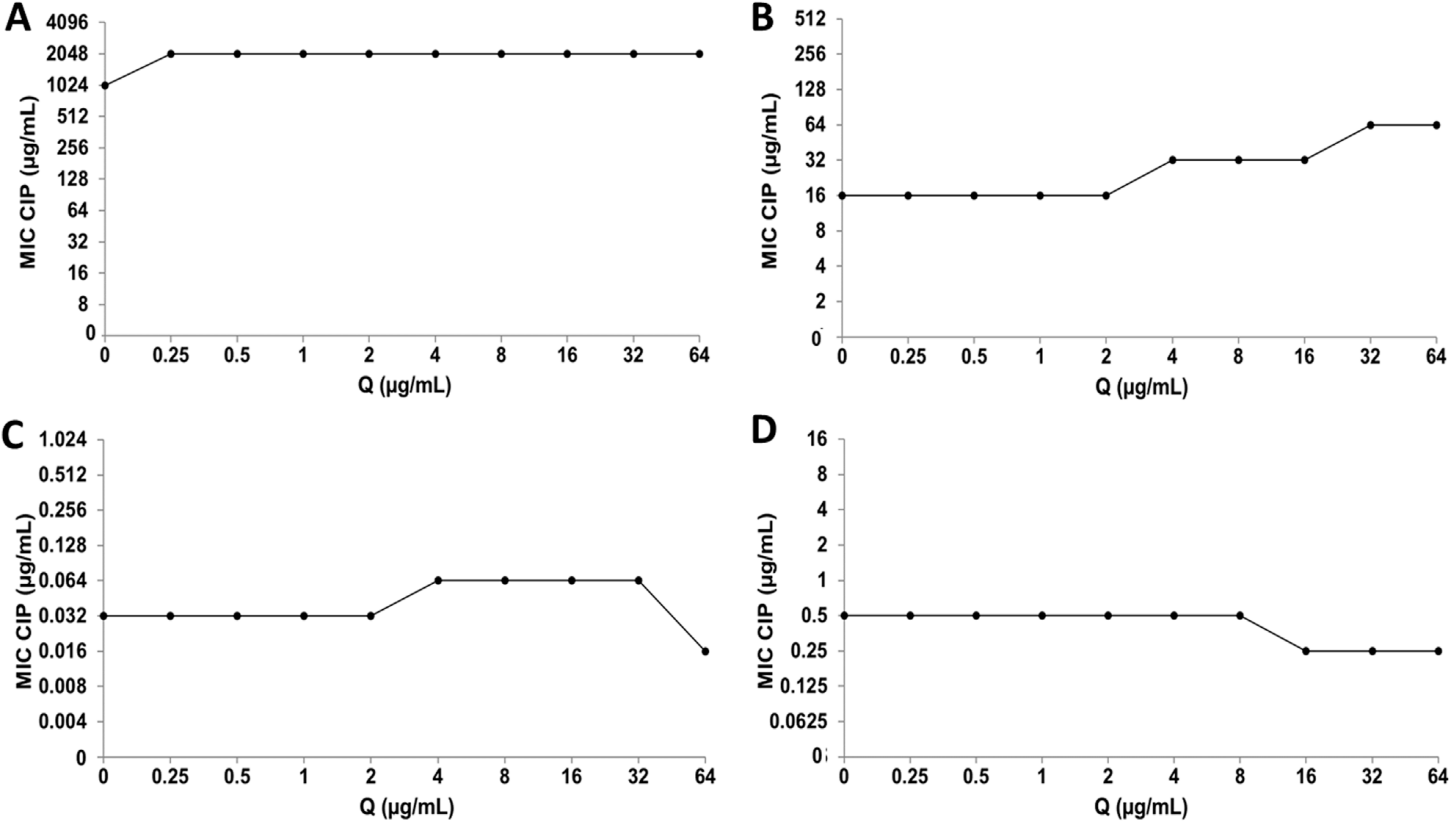
Quercetin (Q) and ciprofloxacin (CIP) interaction by checkerboard technique. **(A)** *Escherichia coli* clinical strain resistant to CIP. **(B)** *Staphylococcus aureus* clinical strain resistant to CIP. **(C)** *Escherichia coli* ATCC 25922. **(D)** *Staphylococcus aureus* ATCC 29213.

Using the checkerboard test, the effect of combining CIP or CMP with Q or LT on the antimicrobial activity of these antibiotics was determined. The combination of CIP and Q for the inhibition of *S. aureus* ATCC 29213 and both *E. coli* strains did not result in changes in the antibacterial activity of CIP (Figures 6A,C,D). However, the CIP+Q combination in the clinical strain of *S. aureus* caused a decrease in bacterial sensitivity to CIP, since the MIC value of the antibiotic increased at one and two dilutions compared to their individual MIC values (Figure 6B). For Q, it was not possible to determine the FICI value, which allows us to evaluate the type of interaction that took place, because experimentally we do not have the value of its individual MIC values (Bustos et al., 2016).

**FIGURE 7:**
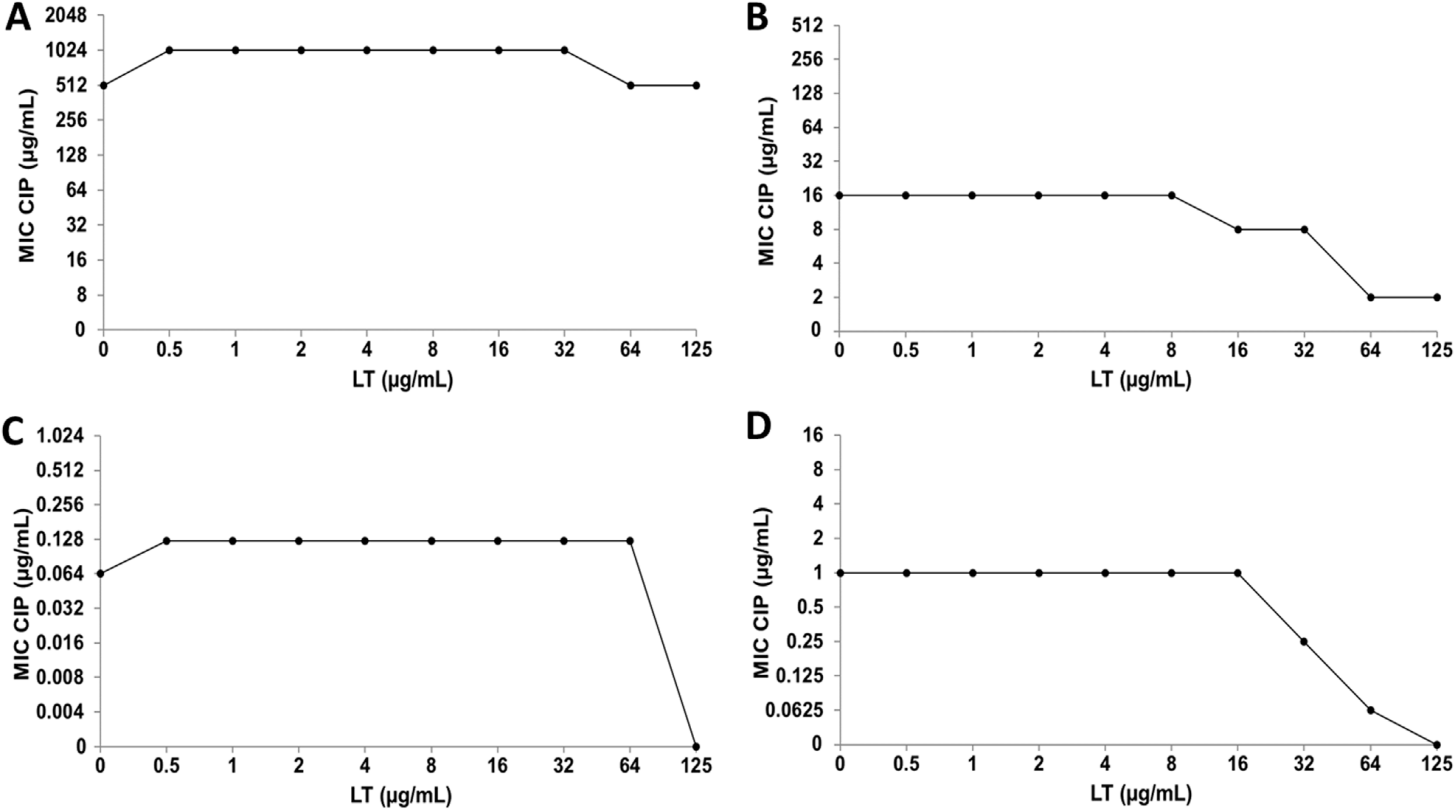
Luteolin (LT) and ciprofloxacin (CIP) interaction by checkerboard technique. **(A)** *Escherichia coli* clinical strain resistant to CIP. **(B)** *Staphylococcus aureus* clinical strain resistant to CIP. **(C)** *Escherichia coli* ATCC 25922. **(D)** *Staphylococcus aureus* ATCC 29213.

When CIP was combined with LT for the inhibition of *E. coli* strains, no changes in the antibacterial activity of CIP were observed (Figures 7A,C), while in the clinical strain of *S. aureus* an increase in bacterial susceptibility to CIP was observed, since it decreased in one and three dilutions of the MIC value (Figure 7B). Furthermore, the combination of CIP+LT for the inhibition of *S. aureus* ATCC increased bacterial susceptibility to CIP, as the MIC value decreased at two and four dilutions compared to the individual MIC values (Figure 7D).

**FIGURE 8:**
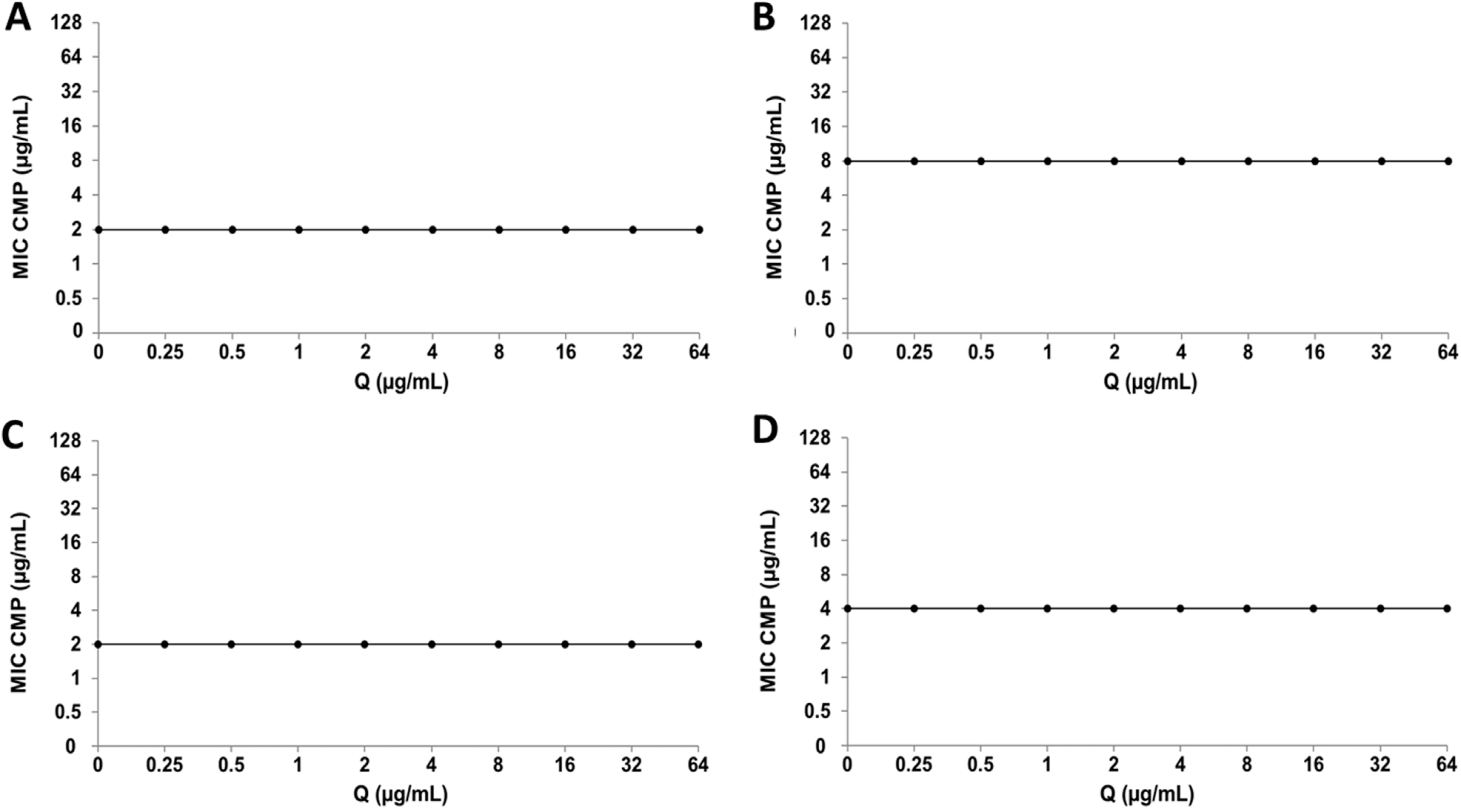
Quercetin (Q) and chloramphenicol (CMP) interaction by checkerboard assay. **(A)** *Escherichia coli* clinical strain resistant to CIP. **(B)** *Staphylococcus aureus* clinical strain resistant to CIP. **(C)** *Escherichia coli* ATCC 25922. **(D)** *Staphylococcus aureus* ATCC 29213.

**FIGURE 9:**
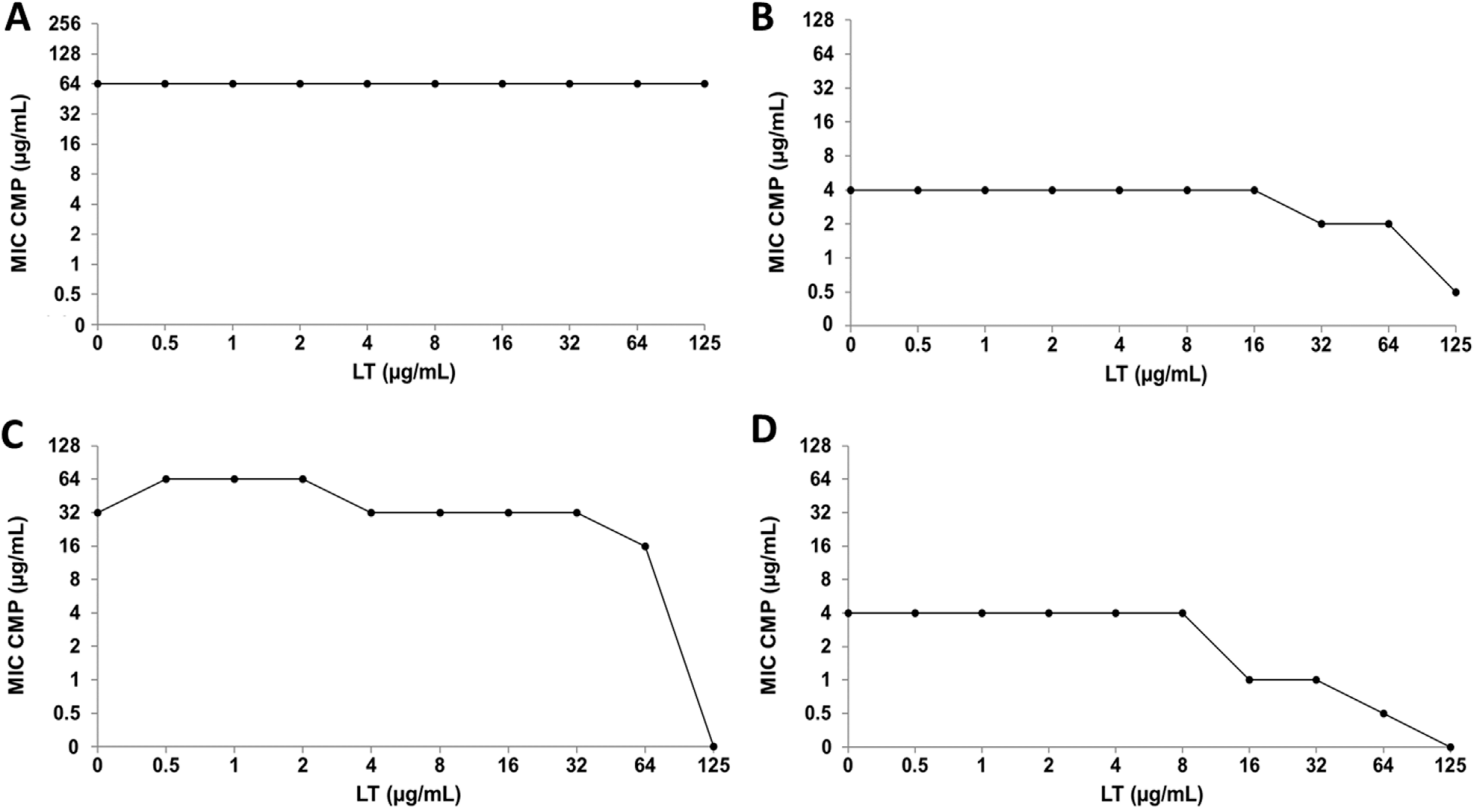
Luteolin (LT) and chloramphenicol (CMP) interaction by checkerboard assay. **(A)** *Escherichia coli* clinical strain resistant to CIP. **(B)** *Staphylococcus aureus* clinical strain resistant to CIP. **(C)** *Escherichia coli* ATCC 25922. **(D)** *Staphylococcus aureus* ATCC 29213.

Regarding CMP, the combination with Q does not generate alterations in the MIC value of the antibiotic in any of the bacterial strains analyzed (Figure 8). On the other hand, in the interaction of CMP with LT, no significant changes were observed in the bacterial sensitivity of the *E. coli* strains (Figures 9A,C), while the clinical strain of *S. aureus* showed an increase in bacterial sensitivity to CMP as the MIC value of CMP decreased by three dilutions compared to their individual MIC values (Figure 9B). Finally, in the CMP+LT combination for the inhibition of *S. aureus* ATCC, an increase in bacterial susceptibility to CMP was observed, since the MIC value decreased in two and three dilutions with respect to its value (Figure 9D).

Because individual MIC values of LT were obtained in the *S. aureus* ATCC strain (Bustos et al., 2018), it was possible to calculate the FICI for the CIP+LT and CMP+LT combinations, giving us information about the type of interaction that occurs in these combinations (Table 3). In the CIP+LT combination, a synergistic effect was observed (FICI = 0.5), showing that the MIC values of CIP and LT were reduced four-fold relative to their individual MIC values. Regarding the CMP+LT combination, synergy was observed with a FICI value of 0.378, where the MIC value of CMP was reduced four-fold and that of LT eight-fold relative to their individual MIC values. Furthermore, an additive effect was observed in the combination of LT with both antibiotics for the inhibition of *S. aureus* ATCC.

**TABLE 3:** Minimal Inhibitory Concentration (MIC), Fractional Inhibitory Concentration (FIC) and FIC Index (FICI) of CIP and CMP in combination with LT in ATCC strains of *E. coli* and *S. aureus*.

*S. aureus* ATCC 29213					
CIP (MIC = 1)			CMP (MIC = 4)		
MICc	FIC_C_	FICI_C+L_	MIC_M_	FIC_M_	FICI_M+L_
0.25	0.25	0.5	1	0.25	0.378
0.062	0.062	0.562	0.5	0.125	0.625

MIC_C_ = MIC of ciprofloxacin in combination with luteolin. FIC_C_ = FIC of ciprofloxacin in combination with luteolin. FICI_C+LT_ = FIC of ciprofloxacin plus FIC of luteolin. MIC_M_ = MIC of chloramphenicol in combination with luteolin. FIC_M_ = FIC of chloramphenicol in combination with luteolin. FICI_M+LT_ = FIC of chloramphenicol plus FIC of luteolin.

## 4 Discussion

Adverse drug reactions that disrupt redox balance and increase ROS represent a significant challenge for safe drug use. Ciprofloxacin is a broad-spectrum fluoroquinolone with a recommended dose of 250 or 500 mg every 12 h in most cases, achieving peak plasma concentrations between 0.5 and 3.7 μg/mL (Sánchez Navarro et al., 2002). Chloramphenicol is a broad-spectrum amphenicol, but has limited use. Although its significant toxicity is known, the World Health Organization (WHO) recommends its use in developing countries due to the lack of more affordable treatments. The recommended dose is 50 mg/kg/day every 6 h, achieving a maximum recommended plasma concentration of 10–25 μg/mL. Since hematological toxicity may be related to serum concentrations, peak concentrations above 25 μg/mL are not recommended (Mendoza-Patiño, 2008). Several studies have shown that the induction of oxidative stress in host cells is associated with the toxicity generated by CIP and CMP. In this study, the ability of CIP and CMP to induce ROS production in human MN and PMN leukocytes was evaluated by testing a range of concentrations, including the plasma concentration of each antibiotic. The results demonstrated that both antibiotics are capable of increasing ROS production only in PMN cells, with the most significant effect in the CMP. This differentiated response between PMN and MN cells to a ROS stimulus had already been described with the antibiotic GEN (Bustos et al., 2016; Bustos et al., 2018) and could be due to differences in the content and response of the main enzymes involved in ROS production, nicotinamide adenine dinucleotide phosphate (NADPH) oxidase and myeloperoxidase, between MN and PMN leukocytes. Regarding CIP-induced intracellular ROS production, the results obtained were consistent with reports showing that CIP at 0.9 μg/mL is capable of generating increased ROS production in human neutrophils (Becerra et al., 2003). In the case of CMP, our results showed an increase in ROS production at concentrations of 0.5 and 16 μg/mL, while at concentrations higher than the recommended plasma concentrations (50 μg/mL), the decrease in ROS production was also accompanied by a decrease in cell viability. This behavior was observed in previous studies where a biphasic response in the percentage of ROS variation was observed with different amounts of CMP; ROS production increased with therapeutic concentrations of antibiotic (13 μg/mL in whole blood and 16 μg/mL in human neutrophils) and decreased with higher concentrations (130–1,300 μg/mL in whole blood and 32 μg/mL in human neutrophils) (Páez et al., 2008; Correa-Salde and Albesa, 2009). The observed decrease in cell viability would explain the reduction in ROS production at toxic concentrations, resulting from excessive oxidative stress.

SOD and CAT enzymes are the most critical endogenous enzymatic defense mechanisms against the toxic effects of ROS. SOD catalyzes the dismutation of superoxide anion to H_2_O_2_, which is transformed into water and oxygen by the CAT enzyme. In PMN leukocytes, CIP increased SOD and CAT activity, while CMP decreased both enzymes. Previous studies with GEN indicated that an increase in the activity of endogenous antioxidant enzymes would occur as a response to counteract increased ROS production, while a decrease in this activity could be due to the rapid consumption and depletion of stored enzymes to eliminate excessive free radical production (Celik and Suzek, 2009; Ademiluyi et al., 2013; Bustos et al., 2016). Therefore, the differences in the effects of CIP and CMP on enzyme activity could be related to the greater ROS induction observed with CMP compared to CIP. Previous studies in human cells with CIP have shown that this antibiotic causes an inhibition of CAT activity in erythrocytes (Qin and Liu, 2013) and a decrease in the levels of the antioxidant enzyme SOD in plasma of patients treated with CIP (Talla and Veerareddy, 2011), while in human fibroblast cultures, no changes in the activities of SOD and CAT are observed after exposure to CIP (Hincal et al., 2003). In contrast to these studies, the results observed in leukocytes exposed to CIP, in which an increase in enzymatic activity was observed, demonstrate the differences in the responsiveness of each cell type to a ROS stimulus and the importance of evaluating each system in particular.

Regarding CMP, previous studies have shown an increase in SOD activity in human neutrophils exposed to concentrations of 4 μg/mL, whereas at 32 μg/mL of CMP, a decrease in enzymatic activity was observed (Páez et al., 2008). In our results, all CMP concentrations evaluated produced a decrease in both SOD and CAT activity in PMN leukocytes.

Several studies have been conducted to evaluate the molecular mechanisms by which CIP and CMP modulate the activity of antioxidant enzymes in various eukaryotic systems. CIP has been reported to increase the activity and protein expression of CAT, SOD isoenzymes 2 and 3, and SOD mRNA levels in *Eisenia foetida* [Lumbricidae] (Wang C. et al., 2018; Yang et al., 2020), decrease the activity and downregulate the gene expression of SOD and CAT in testicular rat tissue (Mokhimar et al., 2023), and bind to the central cavity of CAT with only one binding site and interact with Arg 65, Arg 362, and His 363 through electrostatic forces, causing conformational and functional changes in the CAT enzyme in erythrocytes (Qin and Liu, 2013). On the other hand, a study in *Chamelea gallina* [Veneridae] exposed to CMP demonstrates a decrease in Mn-SOD expression attributed to the adverse effect of CMP on protein synthesis, resulting in a deficiency of the cytosolic precursor of Mn-SOD, with a consequent decrease in Mn-SOD expression (Monari et al., 2008). However, there are no studies to date of the molecular mechanisms involved in the modulation of SOD and CAT by CIP and CMP in human leukocytes. Additional studies are needed to explain the molecular mechanism by which CIP and CMP modify SOD and CAT activity in our system.

When ROS production exceeds the defense capacity of endogenous antioxidant systems, biomolecules such as lipids, DNA, and proteins become targets of oxidative damage. Previous studies with CMP demonstrated increased protein oxidation in whole blood (Correa-Salde and Albesa, 2009), although to date, there are no studies that evaluate the oxidation of macromolecules in blood cells exposed to CIP. In this study, it was observed that, in PMN leukocytes, both CIP and CMP cause an increase in protein oxidation, with the effect of CMP again being greater than that of CIP.

Flavonoids are natural products with significant antioxidant effects that modulate the response to various diseases and conditions associated with redox toxicity. Their antioxidant mechanisms include scavenging ROS, inhibiting oxidases responsible for superoxide anion production, chelating trace metals, and activating antioxidant enzymes (Sarkar et al., 2022; Slika et al., 2022). The flavonoids Q and LT have demonstrated an important protective effect on oxidative stress induced by GEN *in vitro* in human leukocytes and *in vivo* in rat blood (Bustos et al., 2016; Bustos et al., 2018). To extend their protective effect to various classes of antibiotics, their effect on CIP- and CMP-induced oxidative stress was evaluated. The results demonstrated a significant protective effect of Q and LT against CIP- and CMP-induced ROS. Both flavonoids showed IC_50_ values lower than the reference inhibitor (vitamin C), being similar to the effect of Q and LT against ROS induced by CMP (IC_50_ values 3.5 times lower than vitamin C) and greater than the effect of Q with respect to LT in the case of CIP (IC_50_ values of Q three times and IC_50_ values of LT two times lower than vitamin C). Previous results with these antibiotics against GEN-induced ROS in PMN leukocytes showed IC_50_ values of Q similar to vitamin C and LT values slightly higher than the reference inhibitor (Bustos et al., 2016; Bustos et al., 2018). This demonstrates that in human leukocytes, both flavonoids have a greater inhibitory effect on ROS induced by CIP and CMP than against GEN.

Regarding the effect of Q and LT on the activity of endogenous antioxidant enzymes, this work has shown that, regardless of whether there is an increase in enzymatic activity, as in the case of CIP, or a decrease in endogenous antioxidant systems, as in the case of CMP, both flavonoids tend to restore SOD and CAT activities, reaching values similar to those of control cells. In turn, both Q and LT can prevent protein oxidation induced by CIP and CMP, a biomarker of oxidative stress indicative of damage caused by ROS in macromolecules, from reaching values similar to those of control cells. In both the modulating effect of enzyme activity and the prevention of protein oxidation, Q showed a greater effect than LT, as it reached baseline values at all concentrations tested, whereas LT exhibited a more concentration-dependent effect. The greater protective effect observed in Q may be due to its higher antioxidant activity, which, in flavonoids, depends on the arrangement of functional groups in their central structure. The main structural features required for efficient antioxidant activity are an *ortho*-dihydroxy (catechol) structure in ring B, a 2,3-double bond in conjugation with a 4-oxo function in ring C, and hydroxyl groups at positions 3 and 5 (Procházková et al., 2011). Q possesses all the critical structural requirements that determine the antioxidant activity of flavonoids. At the same time, LT lacks the OH group at position 3, which could explain the greater protective effect demonstrated by Q.

The protective effect of Q and LT on PMN leukocytes exposed to CIP and CMP could be due to the significant scavenging activity demonstrated in the ROS inhibition assay, given the ability of flavonoids to transfer electrons and/or hydrogen atoms to hydroxyl, peroxyl, or radical groups of different origins (Procházková et al., 2011). This would cooperate with the endogenous antioxidant defenses SOD and CAT, allowing the recovery of the activity of these enzymes to basal levels and preventing the subsequent protein oxidation induced by these antibiotics. Furthermore, in PMN cells, Q and LT, at the maximum test concentration, can increase the activity of endogenous antioxidant enzymes *per se* (Bustos et al., 2016; Bustos et al., 2018). Therefore, in addition to the direct scavenging activity demonstrated in the ROS inhibition assays, the intrinsic capacity for activation of endogenous antioxidant enzymes could also be involved in their effect against CMP-induced oxidative stress.

The mechanisms involved in the increase in SOD and CAT enzyme activity by LT and Q in combination with CMP, could be due to an increase in the expression of SOD 1, SOD2, and CAT proteins and mRNA, linked by the activation of the Nrf2 pathway, promoting translocation to the nucleus, where it binds to the antioxidant responsive element (ARE) in the promoters of genes encoding SOD and CAT, among others (Wang J. et al., 2018; Xiao et al., 2019; Xu et al., 2019; AL-Megrin et al., 2020; Beken et al., 2020; Xia et al., 2024; Medoro et al., 2025). Furthermore, molecular docking studies indicate that Q formed five hydrogen bonds with Val148, Val7, Lys9, and Asn53 residues in the active site of SOD, implying that it may fit into the binding site of antioxidant enzymes (Bastin et al., 2023). Further studies will be necessary to elucidate the molecular mechanisms underlying the modulation of enzymatic activity by Q and LT in our system.

Since one of the mechanisms of action demonstrated by CIP and CMP would be the oxidative stress production in bacteria (Páez et al., 2010; 2011; Dridi et al., 2015; Masadeh et al., 2020), it was imperative to determine if the antioxidant effect of Q and LT could alter the antibacterial activity of these antibiotics. Furthermore, considering previous studies have demonstrated synergistic effects when LT is combined with GEN, increasing the antibacterial activity of the latter (Bustos et al., 2018), we aimed to investigate whether flavonoids can enhance the antibacterial activity of CIP and CMP. The results demonstrated that Q does not modify the antibacterial activity of CIP and CMP against *E. coli* and *S. aureus* strains, except in the case of the Q+CIP combination against clinical *S. aureus* strains, where an unfavorable trend was observed, as the bacteria’s susceptibility to CIP decreased in the presence of Q. Regarding the combination of LT with CIP and CMP, no changes in the sensitivity of *E. coli* strains were observed, while against *S. aureus* strains, the presence of LT increased the antibacterial activity of CIP and CMP. In the *S. aureus* strain ATCC, in which LT exhibited antibacterial activity on its own, the combination showed antibacterial synergism with both antibiotics; this effect was more pronounced when this flavonoid was combined with CMP. CIP is a bactericidal antibiotic whose antibacterial activity is due to its ability to inhibit the action of DNA gyrase and topoisomerase IV, enzymes responsible for producing negative DNA supercoiling and allowing bacterial DNA replication (Badawy et al., 2021), while the mechanism of action of CMP consists of the inhibition of protein synthesis, due to its ability to bind to the 50S subunit of bacterial ribosomes, thus inhibiting the formation of the peptide bond and subsequent protein synthesis (Rang et al., 2011). On the other hand, studies of the mechanisms by which flavonoids can exert antibacterial activity have suggested three main mechanisms: damage and/or reduction of plasma membrane fluidity, inhibition of nucleic acid synthesis, and inhibition of energy metabolism in bacteria (Cushnie and Lamb, 2011; Echeverría et al., 2017). Therefore, the combination of mechanisms of action through different pathways could explain the synergistic effect observed between LT and the antibiotics CIP and CMP against the reference strain of *S. aureus*. Thus, while both flavonoids showed a strong protective effect against CIP- and CMP-induced oxidative stress, LT also exhibited a potentiating effect on the antibacterial activity of these drugs.

It is important to note that both Q and LT have been reported in the scientific literature as Pan-Assay Interfering Compounds (PAINS). These compounds through various mechanisms, which may question the specificity and reproducibility of the evaluated biological activity (Bolz et al., 2021). However, despite these limitations, the identification of PAINs does not necessarily invalidate the results obtained *in vitro*. Therefore, Q and LT could have a potential therapeutic benefit, warranting further investigation and validation in an *in vivo* model.

Flavonoids may enhance the efficacy of conventional antibiotics, minimize side effects, and/or reduce the required dose. However, the bioavailability, permeability, and water solubility of flavonoids can be low; therefore, new delivery systems are being developed to improve their absorption and efficacy, ultimately achieving better therapeutic results (Salehi et al., 2020; Liu et al., 2025). Furthermore, it is crucial to consider potential antibiotic-flavonoid interactions and conduct an appropriate clinical evaluation before implementing them in clinical practice. In recent years, various combined formulation systems of flavonoids and antibiotics have been evaluated for different uses (Fiore et al., 2024), such as CIP and Q nanofibers for wound healing (Ajmal et al., 2019) or ciprofloxacin and quercetin combination spray dried powder for inhalation (Alhajj et al., 2023). Further research is needed to fully assess the clinical potential of these combinations and determine their best applications.

## 5 Conclusion

In conclusion, both Q and LT showed significant protective effects against CIP- and CMP-induced oxidative stress in human leukocytes, as evidenced by the reduction in ROS production and the restoration of redox balance. While Q demonstrated a more pronounced effect than LT, the latter also potentiated the antibacterial activity of CIP and CMP in *S. aureus* strains, indicating synergistic effects. The clinical applications of these combinations must be investigated by *in vivo* assays proving if the concomitant administration of these flavonoids with CIP and CMP could represent a viable therapeutic strategy to mitigate the oxidative damage induced by these antibiotics in the systemic circulation, thus preventing the clinical consequences associated with reactive species and, in certain cases, increasing the antibacterial activity of these antibiotics. Although significant results have been observed in *in vitro* assays, considering the limitations as mentioned above, further studies *in vivo* are necessary to support the possible efficacy of these combinations.

## Data Availability

The raw data supporting the conclusions of this article will be made available by the authors, without undue reservation.

## Abbreviations

ANOVA, one-way analysis of variance; AOPP, advanced oxidation protein products; ARE, antioxidant responsive element; CAT, catalase; CIP, Ciprofloxacin; CMP, Chloramphenicol; EDTA, ethylenediaminetetraacetic acid; FICI, fractional inhibitory concentration index; GEN, gentamicin; GSH, glutathione; H_2_-DCFDA, 2′,7′-dichlorodihydrofluorescein diacetate; HBSS, Hank’s balanced salt solution; HPLC, High Performance Liquid Chromatography; UPLC-MS/MS, ultra-high-performance liquid chromatography–tandem mass spectrometry; LT, luteolin; MDA, 1,1,3,3 tetraethoxypropane; MIC, minimum inhibitory concentration; MN, Mononuclear; MRM, Multiple Reaction Monitoring; NADPH, Nicotinamide adenine dinucleotide phosphate; NBT, nitroblue tetrazolium; PMN, polymorphonuclear; Q, quercetin; ROS, reactive oxygen species; RNS, reactive nitrogen species; SOD, superoxide dismutase; TBA, 2-thiobarbituric acid; TCA, Trichloroacetic acid; UV–Vis, Ultraviolet-visible; WHO, World Health Organization.
