# Supplementary material for: Evaluation of the protective effect of quercetin and luteolin against ciprofloxacin- and chloramphenicol-induced oxidative stress in blood cells and their impact on the microbiological activity
Source: Front Pharmacol. 2025 Jul 31;16:1626058. doi: 10.3389/fphar.2025.1626058 (PMC12350340; doi:10.3389/fphar.2025.1626058)
Supplement: Supplementary file 1 [file DataSheet1.docx]

**Evaluation of the protective effect of quercetin and luteolin against ciprofloxacin- and chloramphenicol-induced oxidative stress in blood cells and their impact on the microbiological activity**

Pamela Soledad Bustos^1,2^, Javier Echeverría^3*^, Paulina Laura Páez^1,4^, María Gabriela Ortega^1,2*^

^1^ Departamento de Ciencias Farmacéuticas, Facultad de Ciencias Químicas, Universidad Nacional de Córdoba, Córdoba, Argentina.

^2^ Instituto Multidisciplinario de Biología Vegetal (IMBIV-CONICET), Córdoba, Argentina.

^3^ Departamento de Ciencias del Ambiente, Facultad de Química y Biología, Universidad de Santiago de Chile, Santiago, Chile.

^4^Unidad de Investigación y Desarrollo en Tecnología Farmacéutica (UNITEFA-CONICET), Córdoba, Argentina.

**Corresponding authors**

**E-mail address:** Dr. Javier Echeverría (javier.echeverriam@usach.cl); Dra. María Gabriela Ortega (maria.gabriela.ortega@unc.edu.ar)


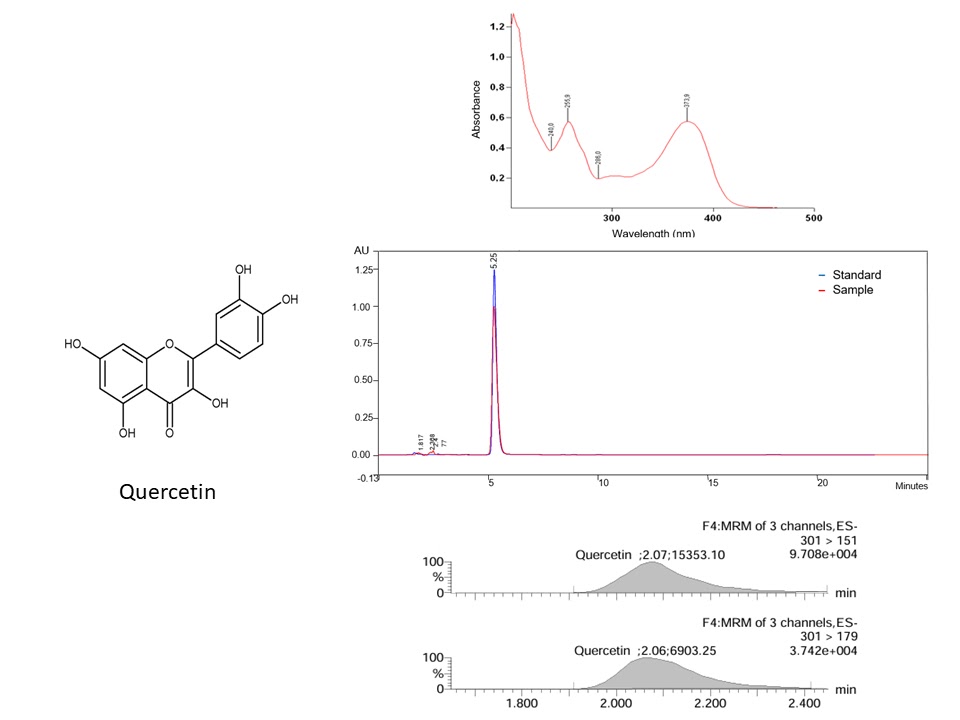


**Figure S1.** Representative Uv-Vis spectra, HPLC chromatograms, and MRM of isolated quercetin (Q).


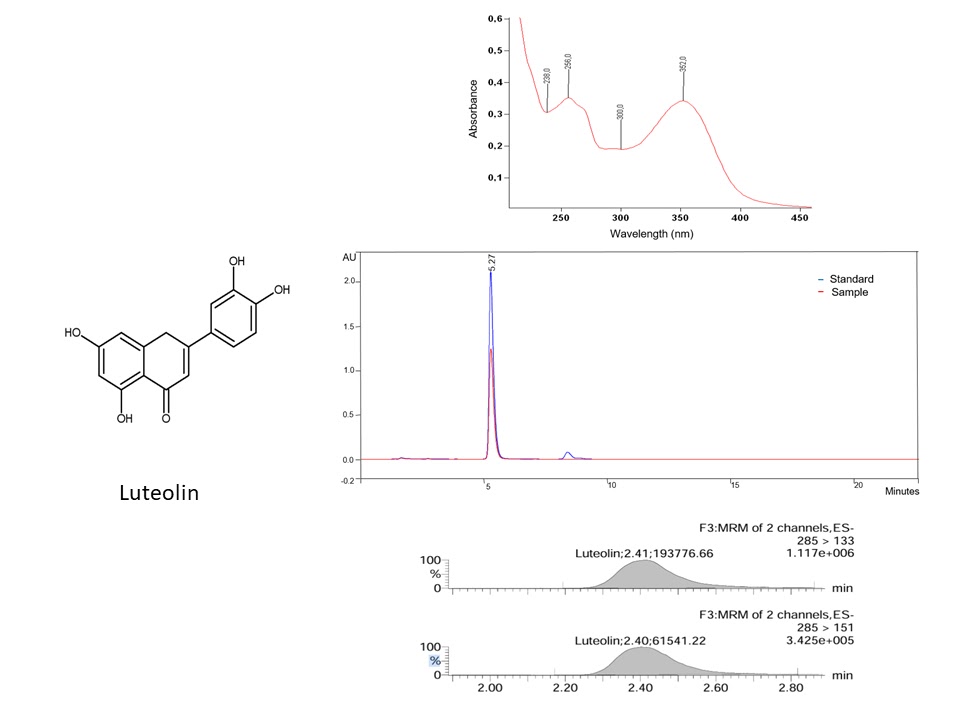


**Figure S2.** Representative Uv-Vis spectra, HPLC chromatograms and MRM of isolated luteolin (LT).
